# Marginal Cell Lymphoma Presenting as a Primary Submandibular Tumor

**DOI:** 10.7759/cureus.11528

**Published:** 2020-11-17

**Authors:** Ani Mnatsakanian, Suzanne Forman, Shant A Korkigian

**Affiliations:** 1 Otolaryngology - Head and Neck Surgery, Ascension Health, Warren, USA; 2 Otolaryngology - Facial Plastic Surgery, Beaumont Health-Farmington Hills, Farmington Hills, USA

**Keywords:** marginal cell lymphoma, non hodgkin's lymphoma, submandibular neoplasm, head and neck tumors

## Abstract

Primary lymphomas of the salivary glands are rare tumors seen in the head and neck. We report the case of a 52-year-old woman with an extranodal marginal cell lymphoma of her submandibular gland, whose primary presenting symptom was a slow swelling of her neck. Fine-needle aspiration of the mass was performed, followed by CT imaging studies, and both tests showed inconclusive results. The tumor was excised and the patient has been monitored with no recurrence to date. This case adds to the diverse differential diagnoses of primary tumors in the head and neck, as early consideration, identification, and management of this etiology is essential for the clinician to ensure the best patient outcomes.

## Introduction

Neoplasms of the salivary gland are rare entities, representing 10% of head and neck tumors [1]. Primary lymphomas of the salivary gland comprise 2% of salivary gland tumors [2]. Extranodal marginal cell lymphoma, a B-cell predominant non-Hodgkin's lymphoma, is an uncommon malignancy of the salivary gland, with scarce literature available regarding the clinical presentation and outcome for these patients. The standard of care for management involves excisional biopsy for diagnosis followed by treatment with radiation therapy.

## Case presentation

**Figure 1:**
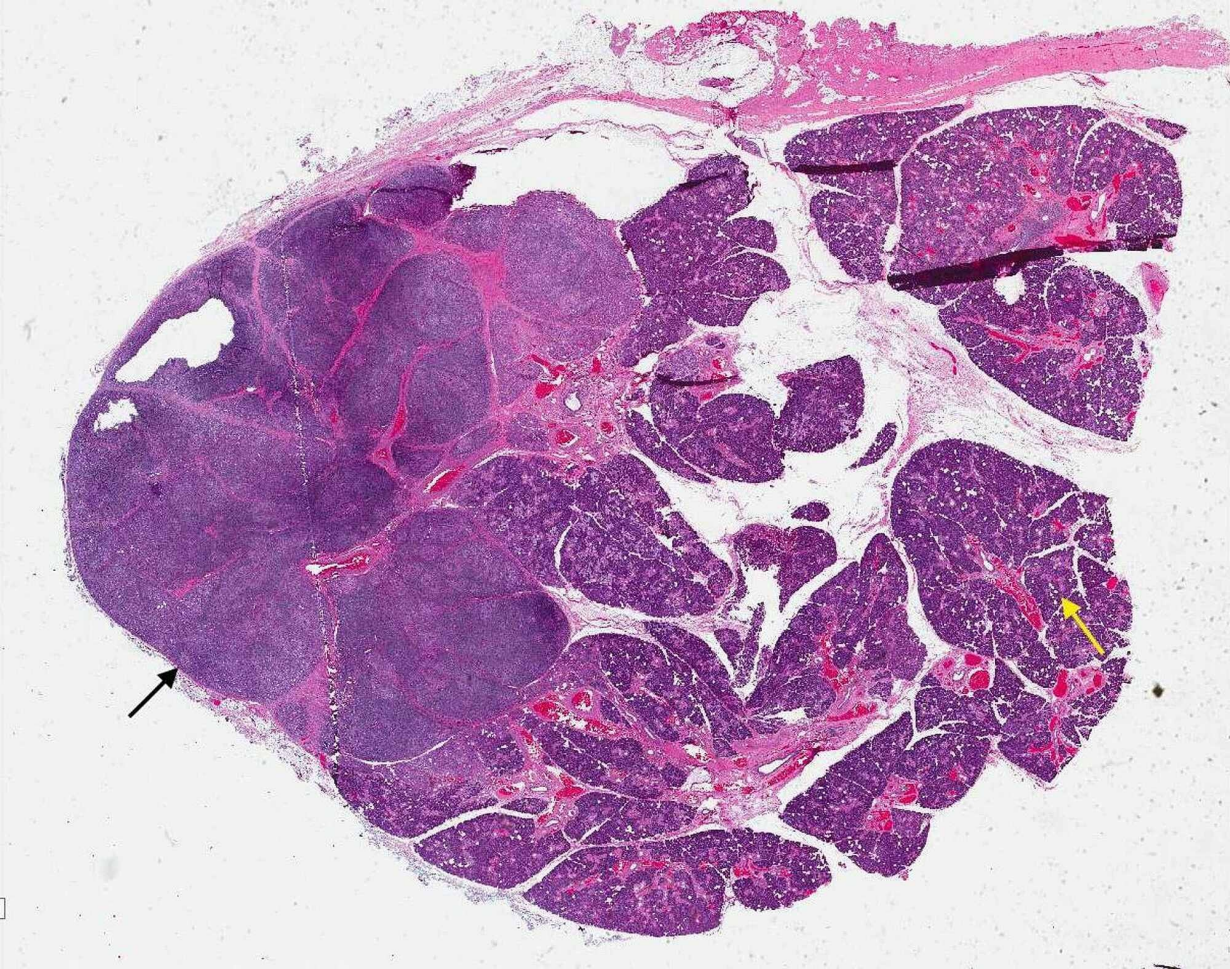
Low-power microscopic examination of the patient’s salivary gland in its entirety The examination shows a normal gland on the left (black arrow) and an abnormal gland with prominent lymphoid infiltrate on the right (yellow arrow)

**Figure 2:**
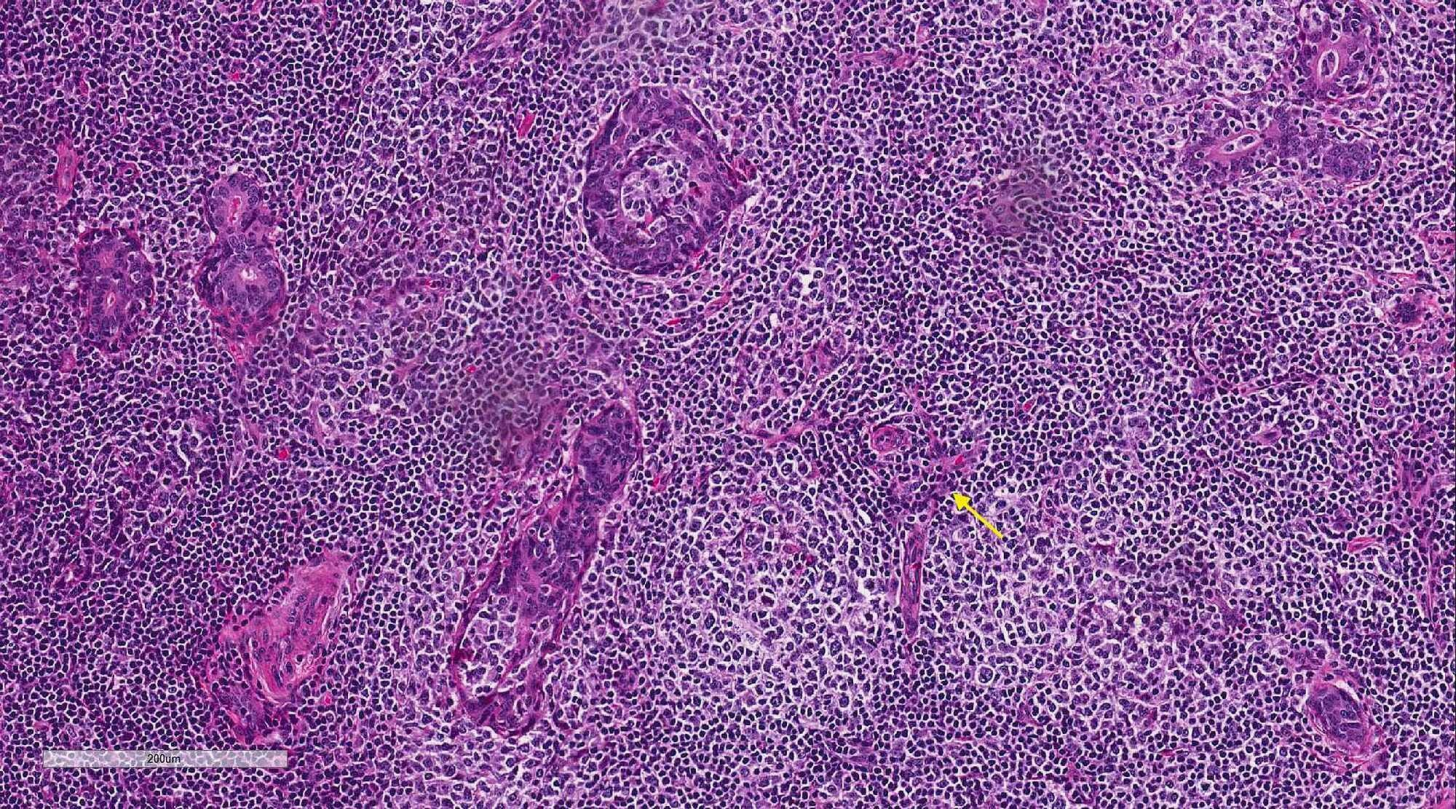
High-powered microscopic examination showing prominent lymphoid infiltrate The infiltrate is composed of variable portions of mature lymphocytes and plasma cells, with multiple areas of infiltration of epithelial structures (yellow arrow)

**Figure 3:**
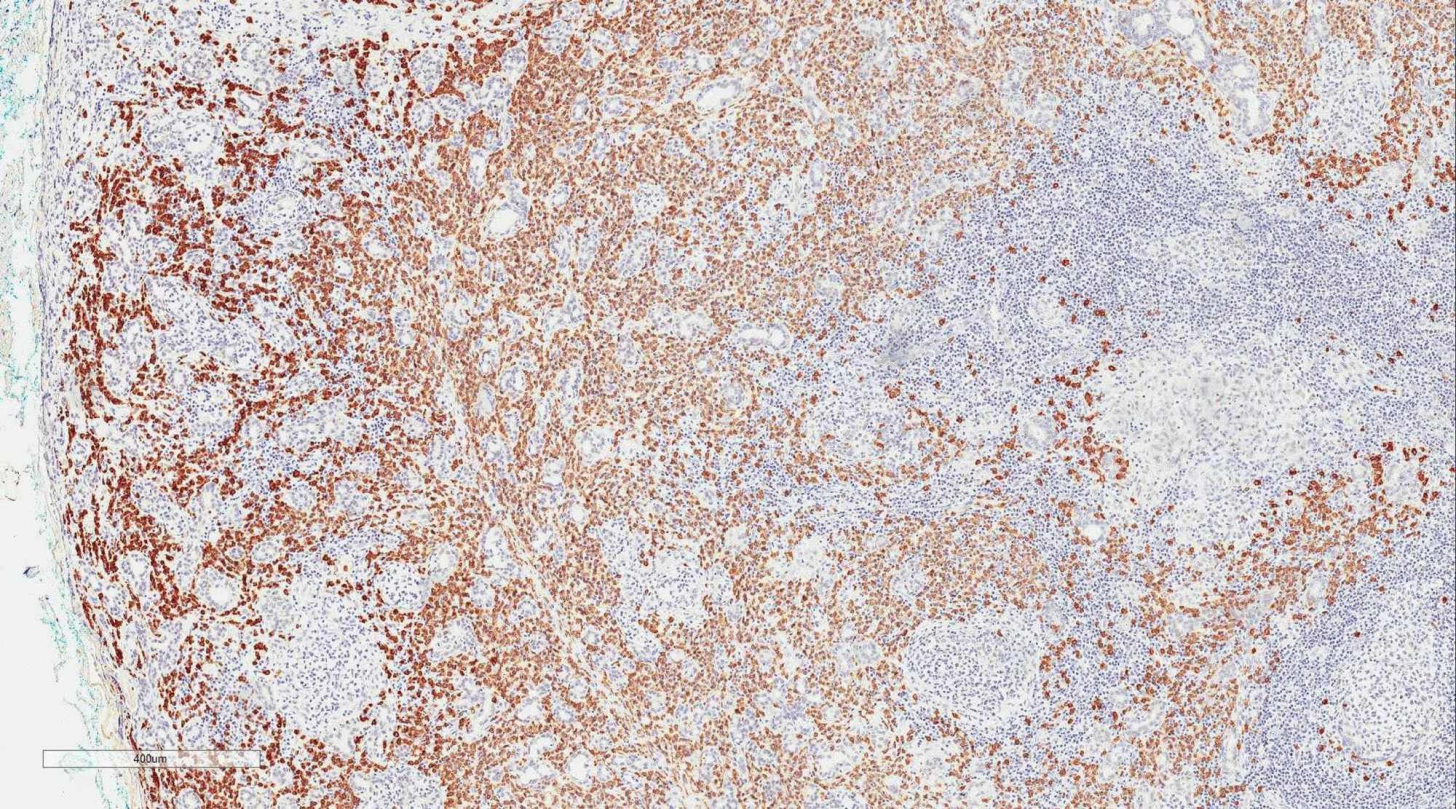
High-powered microscopic examination shows in situ hybridization for kappa and lambda light chain on the plasma cell component of the neoplasm There is a marked increase of lambda-expressing plasma cells in areas, with many fewer kappa cells. This is consistent with a clonal process

A 52-year-old woman presented with an asymptomatic, slowly growing swelling of the left neck. She had undergone an ultrasound, which showed a 1.5 x 0.7 x 1.1 cm hyperechoic mass inside of the left submandibular gland, prompting her to visit an otolaryngologist. Fine-needle aspiration of the mass was performed in the office, which revealed lymphocytes and benign epithelium. Given these results, the differentials for the mass included sialadenitis and lymphoid lesions. She subsequently underwent a CT soft tissue of the neck, which failed to demonstrate the submandibular gland abnormality seen on ultrasound. A decision was made to proceed with surgery to excise the submandibular mass. Final pathology post-excision showed normal salivary gland tissue contrasted with abnormal salivary gland tissue with significant lymphoid infiltrate (Figure 1). The tumor consisted of atypical lymphoid infiltrate of mixed T-cells and B-cells with lambda light-chain predominance and focal granulomatous inflammation (Figures 2, 3). These overall findings favored an extranodal B-cell marginal lymphoma. There were no complications during the surgery. Postoperatively, the patient was referred to radiation oncology and medical oncology for further workup and treatment. The patient is currently being observed and has shown no evidence of recurrence. Postoperative workup and minor salivary gland biopsy did confirm underlying Sjogren’s syndrome for which the patient had been previously asymptomatic.

## Discussion

Extranodal marginal cell lymphoma can affect any extranodal mucosal tissue, with the most common tumor sites being the stomach, other gastrointestinal organs, skin, and respiratory tract [3]. The salivary glands do not typically contain mucosa-associated lymphoid tissue (MALT) but may acquire lymphocytes secondary to chronic inflammatory states [3]. Our patient was a smoker and was found to have Sjogren’s syndrome postoperatively, although she had been previously asymptomatic prior to the diagnosis of submandibular lymphoma. While the entity of extranodal marginal cell lymphoma has been well-described, studies regarding the presentation, clinical course, and treatment outcomes of this disease presenting in the submandibular gland as the primary tumor site are sparse in the literature. The most updated and comprehensive data regarding nongastric, extranodal MALT lymphoma of the salivary glands was described by the Mayo Clinic, with 247 patients in the study cohort [4]. The submandibular gland was the primary site of involvement in 5% of the cases studied [4]. There was no difference between progression-free survival and overall survival in patients receiving radiation, surgery, or chemotherapy as the first-line treatment [4]. The overall 10-year survival rate is 85%, with Sjögren's syndrome serving as a well-established risk factor for the development of this lymphoma [4].

## Conclusions

The treatment protocol for submandibular gland marginal cell lymphoma remains a matter of minimizing long-term treatment-related complications and hence should be individualized to the patient’s specific clinical picture. This represents a diagnostic distinction from most submandibular primary malignancies, in which surgical resection of the tumor with a potential need for neck dissection and/or adjunctive radiation therapy is the first-line treatment. It is important for otolaryngologists to be mindful of this pathological entity in patient counseling and determining treatment regimens regarding submandibular neoplasms.
